# The utility of cerebrospinal fluid white cell count during the prognostic assessment for cryptococcal meningitis patients: a retrospective study

**DOI:** 10.1186/s12879-020-05287-x

**Published:** 2020-08-05

**Authors:** Junyan Qu, Jingwen Jiang, Xiaoju Lv

**Affiliations:** 1 Center of Infectious Disease, West China Hospital, Sichuan University, 37 Guoxue Lane, Chengdu, 610041 China; 2 West China Biomedical Big Data Center, West China Hospital, Sichuan University, Chengdu, China

**Keywords:** Cryptococcal meningitis, Cerebrospinal fluid, White cell count, Clinical outcome

## Abstract

**Background:**

The incidence of cryptococcal meningitis (CM) has gradually increased in recent years. Cerebrospinal fluid (CSF) cytology and cell count are very important for CM on etiology diagnosis and assessment of disease status and therapeutic response. However, the clinical significance of CSF white cell count (WCC) in CM patients is not fully understood. Using longitudinal data of CSF WCC and its relationship with clinical outcomes in CM patients, we aimed to elucidate the clinical significance of this test.

**Methods:**

We retrospectively analyzed the medical records of 150 CM patients admitted to our hospital between January 2008 and December 2018.

**Results:**

CM patients with lower baseline CSF WCC, CSF protein concentration or CD4/CD8 ratio, and those with altered mentation or HIV coinfection were more likely to have poor clinical outcome (*P*<0.05). CM patients with triple therapy during the induction period presented with a better clinical outcome (*P*<0.05). Baseline CSF WCC had a moderate positive correlation with peripheral CD4+ T lymphocyte count (*r* = 0.738, *P* < 0.001) and CD4+ T lymphocyte percentage (*r* = 0.616, *P* < 0.001). The best cut-off value to predict a poor clinical outcome was 40 cells/μL during baseline CSF WCC. The predictive model incorporating longitudinal data of CSF WCC had better sensitivity, specificity, and accuracy than a model incorporating only baseline CSF WCC data.

**Conclusions:**

Our results indicated that baseline CSF WCC and changes in CSF WCC over time could be used to assess the prognosis of CM patients.

## Background

Cryptococcal meningitis (CM), caused by *Cryptococcus* spp*.*, is responsible for significant morbidity and mortality and requires prolonged treatment [1]. It is an opportunistic fungal infection common among human immunodeficiency virus (HIV)-infected patients, particularly in sub-Saharan Africa [2]. Globally, an updated data from 2017 show that annual deaths from CM are about 181,100 [95% confidence interval (CI), 119,400-234,300]. Moreover, 15% (95% CI, 10–19%) of Acquired Immune Deficiency Syndrome (AIDS) patients died from CM [2]. The incidence of CM in HIV-infected patients has decreased in the era of antiretroviral therapy. However, in recent years, CM has been on the rise in HIV-negative hosts with organ transplants, autoimmune disease, immune deficiencies, hematological malignancies, prolonged immunosuppressants treatment, and even in apparently immunocompetent individuals [3]. Recent advances in the care for CM patients and CM prevention, including some new agents and adjunctive immunomodulatory therapies, have the potential to reduce mortality from the disease [4, 5].

Lumbar puncture (LP) is a common procedure in CM patients. Aggressive management of elevated intracranial pressure via LP is beneficial to CM patients [6–8]. Accurate analysis of the cerebrospinal fluid (CSF) profile is essential when diagnosing and managing CM. CSF cellular analysis, India ink staining, CSF Cryptococcal antigen (CrAg) test, fungal culture, and molecular techniques are used to diagnose CM. Many previous studies have shown that high CSF CrAg titer (>1:1024) was an independent risk factor for poor prognosis in CM patients [9, 10]. However, several CrAg measurements are probably unhelpful for CM management because CrAg can still be positive even if the pathogen has been cleared [11]. CSF cytology and cell count can provide quick and cost-effective important information on possible etiologies that could lead to diagnosis and guide immediate therapeutic decisions. More importantly, dynamic changes in CSF cell count could help assess disease status and therapeutic response. Some previous studies have suggested that low baseline CSF white cell count (WCC) was a predictor for poor clinical outcomes in CM patients [10, 12], but other studies showed that CSF WCC was not associated with clinical outcomes [13, 14]. Therefore, the clinical significance of CSF WCC in CM patients is not fully understood, and data on CSF WCC changes over time during antifungal therapy in these patients are rare.

In this retrospective study, we aimed to evaluate the clinical significance of longitudinal CSF WCC data and elucidate its relationship with clinical outcomes in patients with CM who were admitted to a university hospital in China between 2008 and 2018.

## Methods

### Patients

This is a retrospective cohort study was conducted on CM patients admitted to West China Hospital, Sichuan University, Chengdu, Sichuan, China between January 2008 and December 2018. The diagnosis of CM was done based on clinical manifestations and positive CSF culture for *Cryptococcus* or positive CSF India ink staining. Exclusion criteria were age under 14 years, suspected or confirmed co-infection with other pathogens, and those who had received antifungal treatment before admission to our hospital.

### Laboratory studies

Laboratory tests such as routine blood tests, blood biochemistry, and peripheral blood T lymphocyte subsets analysis were carried out. The enzyme-linked immunosorbent assay (ELISA) or chemiluminescence immunoassay was used to detect HIV infection, and positive results were confirmed by western blot (Mp Biomedicals Asia Pacific Pte. Ltd., Singapore). A lymphocyte subset analysis was done by flow cytometry. LP was performed before treatment was initiated. The CSF samples were analyzed immediately for cell count, biochemical parameters, India ink staining, acid-fast smears for tuberculosis (TB), TB DNA analysis, and fungal and bacterial cultures. Latex agglutination (LA) test or lateral flow immunoassay (LFA) was used to detect the CSF CrAg titer. Fungal CSF culture was done in Sabouraud dextrose agar plates. After the initiation of antifungal therapy, CSF examination was performed approximately once a week. All examinations included cell count, biochemical test, India ink staining, and fungal culture.

### Treatment strategies

Cryptococcal disease management was done following the Infectious Diseases Society of America and our country’s guidelines, and based on our experience [6, 7, 15]. Induction therapy for HIV-negative CM patients lasted 6–12 weeks and included amphotericin B (0.5–1.0 mg/kg/day) or amphotericin B liposomes (3.0 mg/kg/day) and 5-flucytosine (100 mg/kg/day). Some patients also received fluconazole (400–800 mg/day) or voriconazole (loading dose of 6.0 mg/kg/12 h, a maintenance dose of 4.0 mg/kg/12 h, and further dose adjustments according to the actual blood concentration). Consolidation therapy was given for more than 6 months, and consisted of fluconazole (400–600 mg/day), voriconazole (200 mg/12 h), or itraconazole (200 mg/12 h), with or without 5-flucytosine. Maintenance therapy and antiretroviral therapy were given to patients with HIV infection. Mannitol or glycerin fructose, frequent lumbar drainage, or lumbar subarachnoid continuous drainage was applied to control high CSF pressure.

### Clinical definitions

Clinical outcomes of patients with CM were evaluated at 2 and 10 weeks after diagnosis or initiation of antifungal therapy. Based on clinical manifestations and laboratory findings, clinical outcomes were divided into good or poor. Patients were assigned to the poor outcome group if they had died or the disease has progressed or relapsed. Disease progression was defined as aggravation of consciousness disorders, fever, headaches, and other clinical manifestations, and persistently positive CSF cultures or India ink staining. Relapse was defined as a situation in which CSF culture or India ink staining has turned negative and then positive again, and symptoms and signs of infection appear again after they have disappeared. A good outcome was defined as a situation in which CSF culture or India ink staining turned negative after being positive, and clinical symptoms have improved.

### Statistical analysis

Statistical analysis was carried out using SPSS for Windows, version 22.0 (IBM Corp., Armonk, NY, USA). Continuous variables were assessed for normality using the Shapiro-Wilk test. Groups were compared using the Student’s *t*-test for normally distributed continuous variables or the Mann-Whitney *U* test for non-normal data. Categorical variables were compared using the Chi-squared test or Fisher’s exact test. Associations between quantitative variables were assessed by Spearman’s rank correlation coefficient (*r*) for non-normal data. An *r*-value greater than 0.8 was considered strong positive correlation; 0.2 < *r* < 0.5 was considered as weak correlation. CSF WCC receiver operating characteristic (ROC) curve was plotted, and the area under the curve (AUC) was calculated using the MedCalc software, version 11.5.1.0 (MedCalc Software, Ostend, Belgium).The AUC was used to evaluate the prognosis for CM patients. A machine learning algorithm (C 5.0 decision tree) was implemented in the R 3.5.1 program for Windows to identify the relationship between dynamic changes in CSF WCC and clinical outcome, and the predictive value of the dynamic changes in CSF WCC for clinical outcome. We split the data into training (70%) and test (30%) datasets while preserving the proportions of the good and poor outcome categories. The training data set was used to build a C5.0 decision tree model. We then repeated 10 times a random leave-one-out cross-validation (LOOCV) test to identify the optimal parameters. The testing dataset was then used in the trained model to predict the outcome. We randomly chose 50 seeds to run our model 50 times. The final model results and the features’ importance analysis results (the importance interval being 0 ~ 100) are the mean values of these 50 runs. A two-tailed *P*-value < 0.05 was considered statistically significant.

## Results

### Patient characteristics

A total of 150 patients with CM (mean age 45.93 ± 15.73 years; 98 males) were admitted to our hospital from January 2008 to December 2018 and were included in this retrospective study. They were categorized into two groups (good and poor outcome groups) according to their clinical outcome. Clinical improvements after 2 and 10 weeks were observed in 93 and 94 CM patients, respectively. Therefore, data assessed after 10 weeks was used for clinical analysis. The demographic and clinical features of these patients are summarized in Table 1. CM was more prevalent between the ages of 31 and 60 years (91, 60.67%) than in the other age groups. Male patients older than 60 years were more likely to have a poor clinical outcome (*P*<0.05). The most common clinical symptoms were headache (136, 90.67%) and vomiting (78, 52.00%). Altered mentation was more common in the poor outcome than the good outcome group (*P*<0.05). The most frequent underlying diseases were HIV infection (32, 21.33%) and immune system disease (16, 10.67%). A higher percentage of patients in the good outcome group (26.60%, 25/94) had no underlying diseases or factors compared to the poor outcome group (12.50%, 7/56; *P*< 0.05).

**Table 1:** Demographic and clinical characteristics in patients with cryptococcal meningitis

Variables	All (*N* = 150) (n,%)	Good outcome (*N* = 94) (n,%)	Bad outcome (*N* = 56) (n,%)	*P*-value
Male	98 (65.33)	58 (61.70)	40 (71.43)	0.288
**Age**				
≤ 30	31 (20.67)	26 (27.66)	5 (8.93)	**0.000**
31–60	91 (60.67)	59 (62.77)	32 (57.14)	
>60	28 (18.67)	9 (9.57)	19 (33.93)	
**Presenting symptoms and signs**				
Headache	136 (90.67)	89 (94.68)	47 (83.93)	**0.029**
Vomiting	78 (52.00)	46 (48.94)	32 (57.14)	0.331
Fever	74 (49.33)	52 (55.32)	22 (39.29)	0.057
Altered mentation	31 (20.67)	12 (12.77)	19 (33.93)	**0.002**
Abnormal vision	29 (19.33)	20 (21.28)	9 (16.07)	0.435
Cough	12 (8.00)	7 (7.45)	5 (8.93)	0.746
Abnormal hearing	9 (6.00)	5 (5.32)	4 (7.14)	0.726
Seizure	7 (4.67)	4 (4.26)	3 (5.36)	1.000
**Underlying diseases or factors**				
HIV infection	32 (21.33)	8 (8.51)	24 (42.86)	**0.000**
Immune system disease	16 (10.67)	12 (12.77)	4 (7.14)	0.413
Hepatobiliary diseases	14 (9.33)	11 (11.70)	3 (5.36)	0.170
Hypertension	13 (8.67)	9 (9.57)	4 (7.14)	0.768
Corticosteroid medication or immunosuppressive drugs	15 (10.00)	9 (9.57)	6 (10.71)	0.822
Diabetes mellitus	12 (8.00)	9 (9.57)	3 (5.36)	0.536
Chronic lung disease	6 (4.00)	4 (4.26)	2 (3.57)	1.000
Renal transplantation	7 (4.67)	4 (4.26)	3 (5.36)	1.000
Tuberculosis	6 (4.00)	1 (1.06)	5 (1.79)	**0.027**
Others	26 (17.92)	15 (15.96)	11 (19.64)	0.564
None	32 (21.97)	25 (26.60)	7 (12.50)	0.042

### Laboratory data

The laboratory tests results in patients with CM are shown in Table 2. The baseline CSF WCC and protein concentration in the poor outcome group were lower than the good outcome group (*P* < 0.05). The positive rate of CSF India ink staining in the poor outcome group (54, 96.43%) was higher than in the good outcome group (66, 70.21%; *P* < 0.05). There was no difference in CSF glucose or chloride levels and the positive rates of CSF fungal culture between the two groups. The percentage of serum CD4+ T lymphocyte (11.97% vs. 31.35%, *P* < 0.001) and CD4/CD8 ratio (0.10 vs. 1.19, *P* < 0.001) were lower in the poor outcome group than in the good outcome group.

**Table 2:** Laboratory data in patients with cryptococcal meningitis

Variables	All (*N* = 150)	Good outcome (*N* = 94)	Bad outcome (*N* = 56)	*P*-value
**CSF**				
WCC (cells/μl), Median (IQR)	60.00 (10.00–140.00)	90.00 (46.00–180.00)	10.00 (0.00–40.00)	**0.000**
Total protein (mg/dL), Median (IQR)	0.81 (0.52–1.14)	0.87 (0.61–1.23)	0.65 (0.41–0.91)	**0.002**
Glucose (mmol/L), Median (IQR)	1.95 (0.87–2.73)	1.88 (0.80–2.66)	1.99 (0.97–2.96)	0.728
Chloride (mmol/L), (mean ± SD)	118.75 ± 6.14	118.62 ± 5.70	118.96 ± 6.85	0.345
Positive India ink stain(%)	120 (80.00%)	66 (70.21%)	54 (96.43%)	**0.000**
Positive CSF fungal culture(%)	116 (77.33%)	75 (79.79%)	41 (73.21%)	0.421
Positive India ink stain & fungal culture(%)	96 (64.00%)	57 (60.64%)	39 (69.64%)	0.295
**Serum**				
Albumin(g/L), (mean ± SD)	39.73 ± 4.81	40.43 ± 4.33	37.98 ± 5.56	0.025
WBC (× 10^9^/L), (mean ± SD)	8.35 ± 3.46	8.54 ± 3.20	8.01 ± 3.88	0.364
Hemoglobin (g/L), (mean ± SD)	124.49 ± 19.66	127.95 ± 20.51	120.02 ± 19.67	0.022
CD4^+^ T lymphocyte (%),(mean ± SD)^a^	25.14 ± 16.75	31.35 ± 14.40	11.97 ± 13.58	**0.000**
CD8^+^ T lymphocyte (%),(mean ± SD)^a^	34.52 ± 16.80	29.61 ± 13.61	44.92 ± 18.32	**0.000**
CD4/CD8 ratio (%), Median (IQR)^a^	0.93 (0.20–1.57)	1.19 (0.70–1.76)	0.10 (0.03–0.83)	**0.000**

^a^47 missing values, *CSF* cerebrospinal fluid, *WCC* white cell count, *WBC* white blood cell

### Treatment strategies and clinical outcomes

A total of 134 CM patients received antifungal treatment after diagnosis. The remaining patients (16/150, 10.67%) did not receive antifungal treatment because their disease had rapidly progressed to death before such treatment could be initiated or because they were abandoned by their family members and were discharged voluntarily. Treatments for the other patients were categorized as monotherapy, two-drug combination, or triple therapy. Most patients (81/150, 54.00%) were treated by the two-drug combination, but patients receiving the triple therapy had a better clinical outcome (*P* < 0.05), as shown in Table 3.

**Table 3:** Therapeutic regimen of 150 patients with cryptococcal meningitis

Treatment	All (*N* = 150) (n,%)	Good outcome (*N* = 94) (n,%)	Bad outcome (*N* = 56) (n,%)	*P*-value
No treatment	16 (10.67)	0 (0.00)	16 (28.57)	**0.000**
Monotherapy	11 (7.33)	3 (3.19)	8 (14.29)	
Two-drug combination	81 (54.00)	54 (57.45)	27 (48.21)	
Triple therapy	42 (28.00)	37 (39.36)	5 (8.93)	
**Antifungal treatment**				
AmB	9 (6.00)	3 (3.19)	6 (10.71)	0.079
FCZ	2 (1.33)	0 (0.00)	2 (3.57)	0.138
AmB + 5FC	59 (39.33)	40 (42.55)	19 (33.93)	0.296
AmB + FCZ	9 (6.00)	4 (4.26)	5 (8.93)	0.295
AmB + VCZ	3 (2.00)	1 (1.06)	2 (3.57)	0.556
FCZ + 5-FC	4 (2.67)	3 (3.19)	1 (1.79)	1.000
VCZ + 5-FC	6 (4.00)	6 (6.38)	0 (0.00)	0.084
AmB + FCZ+ 5-FC	15 (10.00)	13 (13.83)	2 (3.57)	0.050
AmB + VCZ+ 5-FC	27 (18.00)	24 (25.53)	3 (5.36)	**0.002**

*AmB* amphotericin B, *FCZ* fluconazole, *VCZ* voriconazole, *5-FC* 5-flucytosine

### Associations with baseline cerebrospinal fluid white cell count

**Fig. 1:**
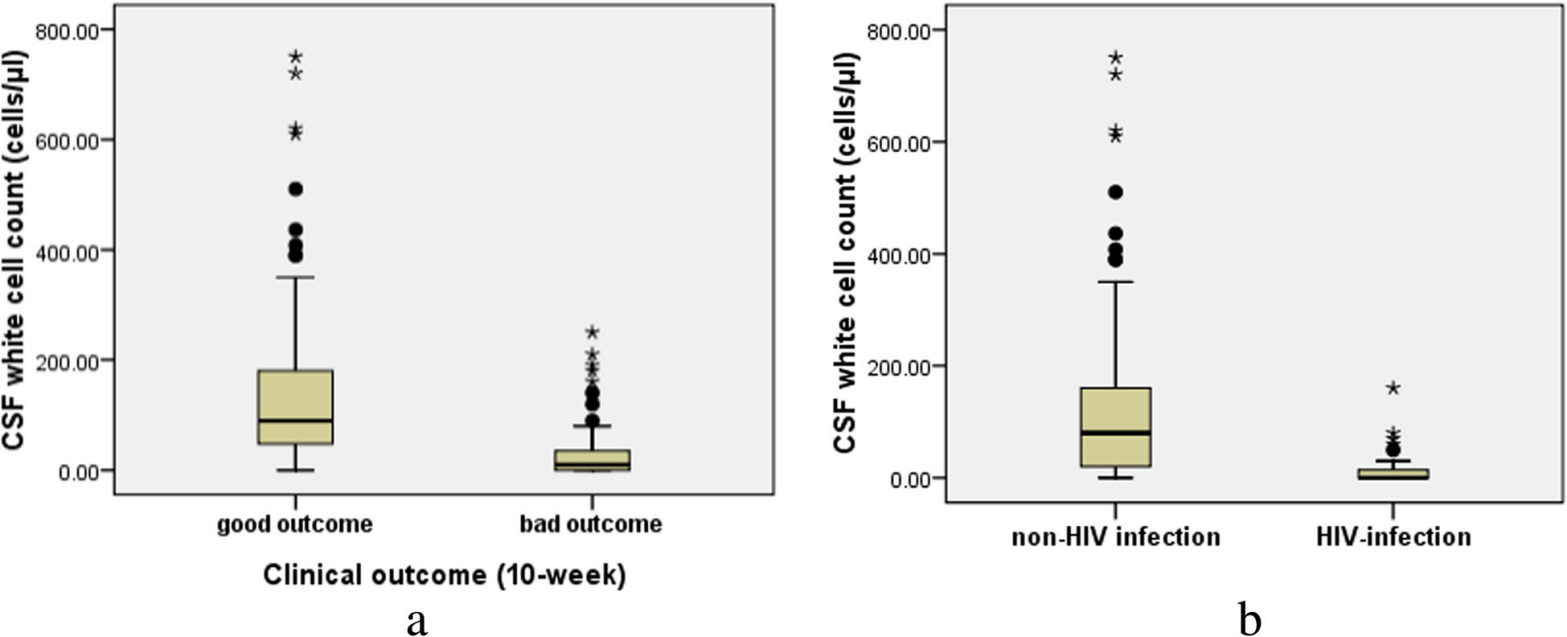
Baseline cerebrospinal fluid white cell count (CSF WCC) in cryptococcal meningitis patients. **a** CSF WCC in the good outcome group was higher than in the poor outcome group (*P* < 0.001); **b** CSF WCC in the non-HIV infection group was higher than the HIV-infection group (*P* < 0.001)

The CM patients were divided in two ways; one division was based on their clinical outcomes, and the other based on their HIV-infection status. As can be seen in Fig. 1, the baseline CSF WCC in the good outcome group (90 vs. 10 cells/μL, *P* < 0.001) and in the non-HIV infection group (80 vs. 0 cells/μL, *P* < 0.001) were higher than in the control group (*P* < 0.01).

### Relationship of baseline cerebrospinal fluid white cell count and T lymphocyte subsets in the peripheral blood

**Fig. 2:**
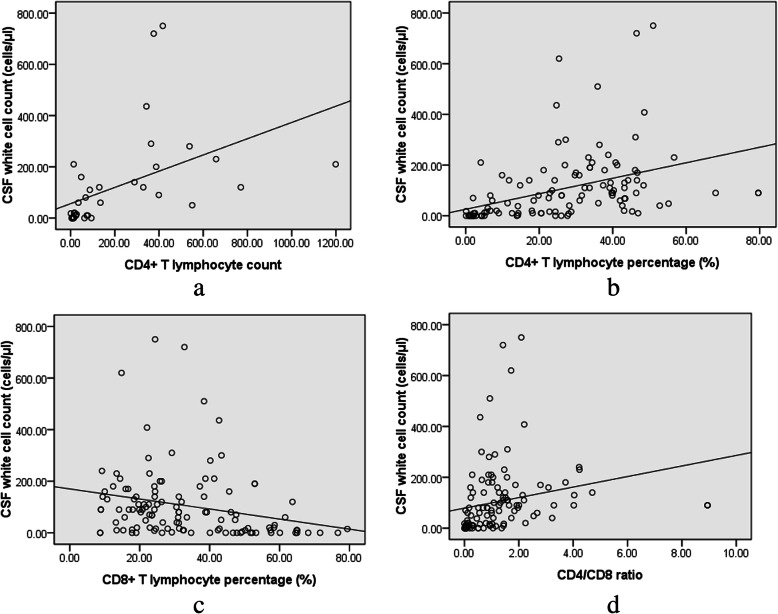
Relationship between cerebrospinal fluid white cell count (CSF WCC) and peripheral T lymphocyte subsets. Spearman correlation analysis was used to study the association between CSF WCC and peripheral CD4+ T lymphocyte count, CD4+ T lymphocyte percentage, CD8+ T lymphocyte percentage, and CD4/CD8 ratio in cryptococcal meningitis patients. **a** CSF WCC was positively correlated with peripheral CD4+ T lymphocyte count (*r* = 0.738, *P* < 0.001), **b** CD4+ T lymphocyte percentage (*r* = 0.616, *P* < 0.001), and (**d**) CD4/CD8 ratio (*r* = 0.592, *P* < 0.001). **c** CSF WCC had a weak negative correlation with CD8+ T lymphocyte percentage (*r* = − 0.364, *P* < 0.001). Forty-seven values of CD4+ T lymphocyte percentage, CD8+ T lymphocyte percentage, and CD4/CD8 ratio were missing

Results of CD4+ T lymphocyte count in the peripheral blood were obtained in 35 cases. The correlations between CSF WCC and CD4+ T lymphocyte count, CD4+ T lymphocyte percentage, CD8+ T lymphocyte percentage, and CD4/CD8 ratio were analyzed. CSF WCC and CD4+ T lymphocyte count were closely correlated (*r* = 0.738, *P* < 0.001), as shown in Fig. 2.

### ROC curve of CSF WCC for prognosis evaluation

**Fig. 3:**
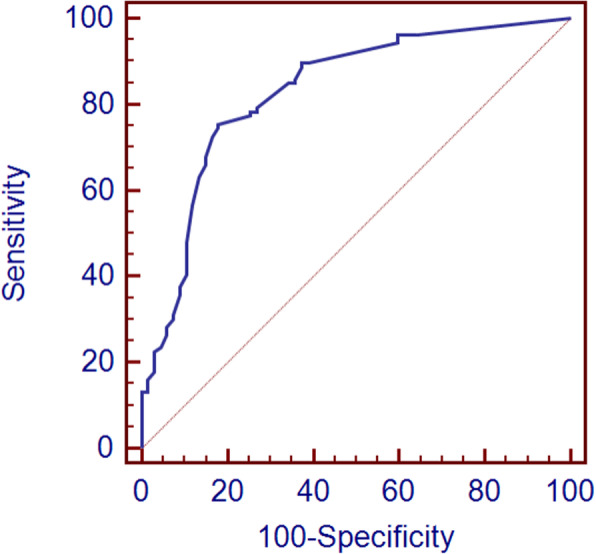
Receiver operating characteristic (ROC) curve for cerebrospinal fluid white cell count (CSF WCC) as a means to evaluate the prognosis of patients with cryptococcal meningitis. The area under the curve (AUC) for CSF WCC was 0.831 [95% confidence interval (CI) 0.766–0.883]

Figure 3 depicts the ROC curve of CSF WCC for prognosis evaluation in patients with CM. The AUC of CSF WCC was 0.831 (95% CI: 0.766–0.883; *P* < 0.001). The most effective cutoff value of CSF WCC for evaluating poor clinical outcomes in CM patients was 40 cells/μL, for which Youden’s index was 0.58.

### The relationship between dynamic changes in CSF WCC and clinical outcome

**Fig. 4:**
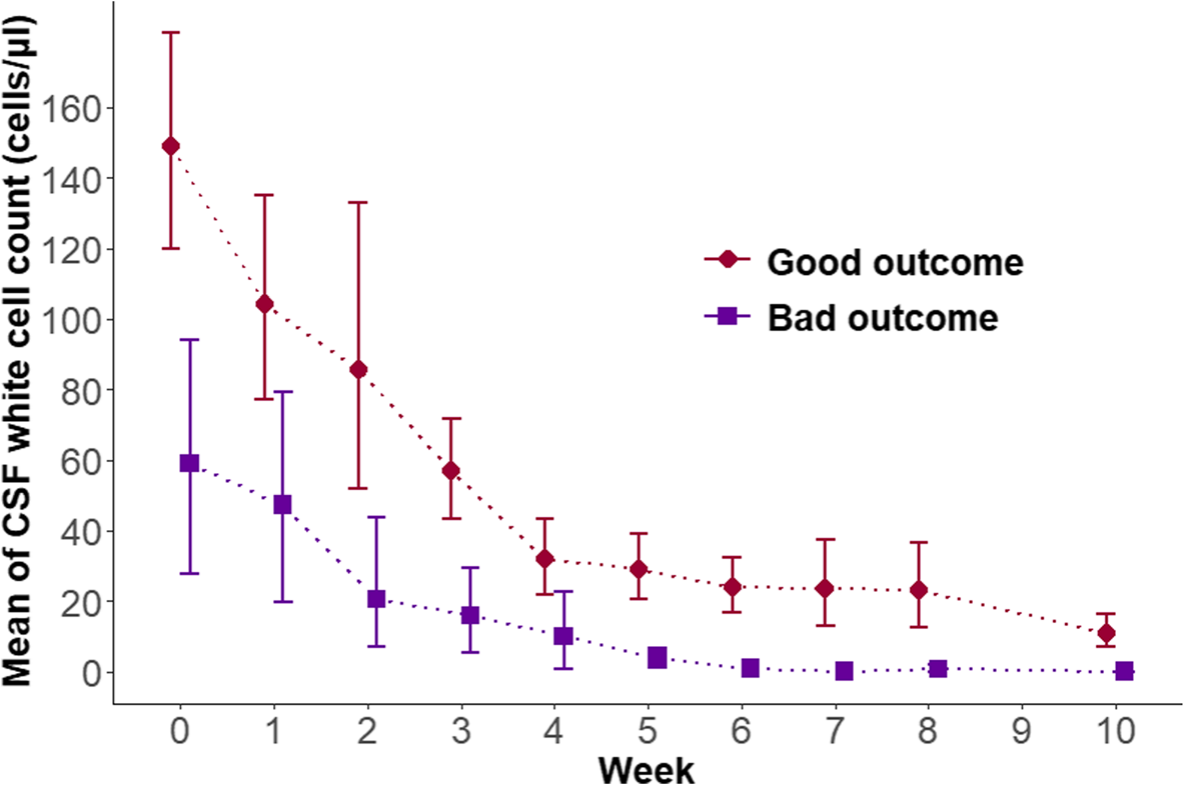
The dynamic changes of cerebrospinal fluid white cell count (CSF WCC) at baseline (week 0) and through antifungal therapy (weeks 1–10) in patients with cryptococcal meningitis (CM). CM patients in the good outcome group had higher CSF WCC in weeks 0–6 and week 10 than those in the poor outcome group (*P* < 0.05). Missing data in each group and each week were not used in Figure 4. No CSF WCC records were available in week 9

The dynamic changes of CSF WCC are shown in Fig. 4. The CSF WCCs were higher among patients in the good outcome group on weeks 0, 1, 2, 3, 4, 5, 6, 10 (*P* < 0.05). The counts decreased faster in this group compared to the poor outcome group. Two predictive models (C5.0 decision tree), using the caret package, revealed the relationship between dynamic changes in CSF WCC and the clinical outcome. The covariables used to predict the outcome in both models were sex, age, Charlson comorbidity index, hemoglobin concentration, peripheral WBC count, neutrophil concentration, treatment strategies, CSF protein concentration, CSF chloride concentration, CSF India ink staining, and CSF culture. Model 1 only used CSF WCC at week 0, while model 2 used CSF WCCs at weeks 0 to 10. The sensitivity, specificity, accuracy, and AUC of model 2 were superior to those of model 1. In both models, CSF WCC at week 0 made a great contribution to predicting the clinical outcome (importance: 92.4 in model 1, 91.5 in model 2). In model 2, the importance of CSF WCCs from week 1 to week 10 were all greater than zero (Table 4).

**Table 4:** Prediction models for clinical outcome in patients with cryptococcal meningitis

Items	Model 1 (week 0)	Model 2 (week 0-week 10)
Sensitivity	0.8828571	0.9035714
Specificity	0.6950000	0.7100000
Positive prediction value	0.8410332	0.8505685
Negative prediction value	0.7917769	0.8306219
Accuracy	0.81454545	0.83318182
AUC	0.8832813	0.8985045
**The importance of variables to the predictive model**		
Treatment (no treatment)	98.00	97.7358
Positive CSF ink stain	91.491	92.6038
Age	89.6036	88.6226
Charlson index	85.5472	80.8676
Treatment (monotherapy)	81.434	86.0568
Treatment (triple therapy)	79.3774	81.1326
Hemoglobin	76.1708	76.982
Negative CSF fungal culture	72.9062	73.5666
Neutrophile	68.3962	60.7552
CSF proteins	67.6798	57.0194
White blood cell	67.4152	49.2082
Male	64.245	58.2074
**CSF WCC**		
week 0	92.3776	91.472
Week 1	NA	15.6604
Week 2	NA	21.1326
Week 3	NA	10.2644
Week 4	NA	7.2828
Week 5	NA	17.17
Week 6	NA	14.1516
Week 7	NA	5.4718
Week 8	NA	3.7928
Week 10	NA	22.095

*CSF* cerebrospinal fluid, *WCC* white cell count, *NA* Not applicable. Only variables whose importance was greater than zero were displayed

## Discussion

Cryptococcal meningitis is the most common life-threatening fungal meningitis, so it is important to recognize its clinical characteristics and explore new methods for early diagnosis and prognostic assessment. This study found that older male patients with CM (≥ 60 years) had the worst clinical outcome. This finding is similar to previous research results [16]. The immune function in older individuals shows the characteristics of immune senescence, as it declines and weakens the ability of older people to respond to infection [17]. This might be one of the reasons for the poor prognosis in older CM patients. Headache, fever, vomiting, and altered mentation were common clinical manifestations of CM in our patients. Many previous reports have also found similar results and showed that altered mentation was an independent indicator of poor outcomes for CM patients [9, 18]. A higher proportion of patients in the good outcome group presented with headache in this study. This finding might be because some patients in the poor outcome group suffered from disturbances to their consciousness and thus could not complain about their sufferings. This study also suggests that CM patients with HIV or tuberculosis infection were associated with worse outcomes. An important characteristic of these patients is their impaired cellular immune function [19, 20]. Such impairment might reduce the recruitment of macrophages and neutrophils to the sites of infection, thereby reducing *Cryptococcus* clearance [21].

CM patients with lower baseline CSF WCC and protein level had poor clinical outcomes. Several previous studies have also found that CM patients with lower CSF WCC had a lower survival rate [22, 23], but other results suggested that baseline CSF WCC is not associated with mortality in patients with HIV-associated CM [13]. Lower CSF WCC indicates a lower intensity of the inflammatory response, thus affecting the clinical outcome. A weak inflammatory response in the central nervous system (CNS) of CM patients with lower CSF protein level might lead to a decrease in the transport of antifungal drugs to the CNS [24]. In this study, a high positive rate of CSF India ink staining was found in the poor outcome group. Positive India ink staining often signifies a high fungal burden, which indicates a poor clinical outcome [13, 25]. CD4+ T lymphocyte percentage and CD4/CD8 ratio in the serum were significantly lower in the poor outcome group. One study examined the cryptococcal-specific peripheral CD4+ T-cell response in HIV-associated CM patients. The researchers found that these cells could produce macrophage inflammatory protein 1α, interferon γ (IFN-γ), and tumor necrosis factor α (TNF-α). These results show that when the IFN-γ/TNF-α-producing CrAg-specific CD4+ T-cell response was missing, CSF lymphocyte count declined, the proinflammatory CSF cytokine response was attenuated, and a higher mortality rate was recorded [26].

Nowadays, options for treating CM remain limited. All patients without access to treatment had a poor clinical outcome. The best clinical outcome was of patients with triple therapy. Therefore, early diagnosis and treatment are very important to reduce the mortality of CM patients. Amphotericin B, combined with 5-flucytosine, remains the most commonly used induction therapy regimen for CM, but its therapeutic efficacy is unsatisfactory. One earlier research found that in HIV-associated CM patients, a combined treatment of amphotericin B and 5-flucytosine could more rapidly clear *Cryptococcus* from the CSF than amphotericin B alone, amphotericin B with fluconazole, or triple therapy [27]. A recent study in CM patients showed that triple therapy that combines amphotericin B, 5-flucytosine, and fluconazole provides superior clinical effectiveness when used from treatment initiation. This triple therapy was superior to the combination therapy of amphotericin B and flucytosine. Importantly, there was no difference between the treatment groups in the incidence of adverse events [28]. Zhao et al. also found a significantly higher response rate in the triple combination group than in the two-drug combination group for salvage therapy in HIV-uninfected CM patients [29]. Triple therapy used during the induction period in CM patients could reduce fungal burden and mortality, so it might be considered as an alternative treatment for these patients.

In this study, we observed lower CSF WCC in the poor outcome group and the HIV-infected group. In addition, CSF WCC was positively correlated with peripheral CD4+ T lymphocyte count, CD4+ T lymphocyte percentage, and the CD4/CD8 ratio. A previous study found a positive correlation between CSF and blood CD4+ counts. Lower numbers of CSF T cells and NK cells, and lower expression of the neutrophil activation marker CD66b26 were also observed in CM patients with a high fungal burden [29]. Lower CSF WCC might indicate a higher fungal burden in the CSF. Patients with higher CSF fungal burden tend to have lower concentrations of proinflammatory cytokines (IL-6, IFN-γ, and TNF-α) in their CSF [30, 31]. This lower cytokine level is probably because of the high concentration of glucuronoxylomannan, the main capsular polysaccharide of *Cryptococcus*. In such patients, the CNS inhibits the recruitment of immune cells into the CSF [32]. Our study also found a baseline CSF WCC cutoff value of 40 cells/μL, below which poor prognosis could be predicted for the CM patients. In some low- and middle-income countries, many hospitals with limited health care resources cannot carry out laboratory tests such as CrAg and cellular immune function, but CSF cytology can be performed in almost every hospital. Therefore, the baseline cutoff value for CSF WCC might help many clinicians around the world assess the prognosis of their CM patients. Analysis using the R program revealed that beyond the baseline CSF WCC, CSF WCC changes over time might be an even more sensitive, specific, and accurate way to predict clinical outcomes. Few publications have assessed the association between CSF WCC changes over time with the prognosis of CM patients. A previous study showed that follow-up CSF cell count could not be used to monitor the efficacy of antifungal therapy in patients with cryptococcal meningoencephalitis [14]. That study, however, included only 21 patients. Our study results might be less biased because of the considerably larger number of patients. We found that decreased CSF WCC was associated with effective antifungal therapy. Combined baseline CSF WCC and CSF WCC changes over time could provide better estimates for the prognosis of CM. The use of such a combined assessment might help clinicians to efficiently manage CM patients and closely monitor those with poor prognosis.

This study has some limitations. It is a retrospective study in a single hospital, and only part of the patients had data on CD4+ T lymphocyte count. However, this is the single-center study with the largest number of cases. Some tests could not be performed in every patient during diagnosis and treatment, which is more consistent with clinical practice. Further multicenter studies involving primary medical care settings, still need to be performed.

## Conclusions

This study shows that CM patients with lower baseline CSF WCC, lower CD4+ lymphocyte count, and altered mentation as well as older male CM patients (≥ 60 years) were more likely to have a poor clinical outcome. Triple therapy during the induction period might be an alternative treatment approach for CM patients with poor prognosis. Baseline CSF WCC had a moderate positive correlation with peripheral CD4+ T lymphocyte count. Baseline CSF WCC below 40 cells/μL might indicate poor prognosis in CM patients. Baseline CSF WCC, combined with CSF WCC changes over time, could better predict the prognosis of CM. More aggressive treatment and effective management should be given to patients with poor prognosis.

## Data Availability

All data generated or analyzed during this study are included in this published article. The datasets used and/or analysed during the current study are available from the corresponding author on reasonable request.

## Abbreviations

CM: Cryptococcal meningitis
CSF: Cerebrospinal fluid
WCC: White cell count
HIV: Human immunodeficiency virus
AIDS: Acquired immune deficiency syndrome
LP: Lumbar puncture
CrAg: Cryptococcal antigen
TB: Tuberculous
ELISA: Enzyme-linked immunosorbent assay
LA: Latex agglutination test
LFA: Lateral flow immunoassay
ROC: Receiver operating characteristic
AUC: Area under the curve
LOOCV: Leave-one-out cross-validation

## References

[1] Chen M, Xu Y, Hong N, Yang Y, Lei W, Du L (2018). Epidemiology of fungal infections in China. Front Med.

[2] Rajasingham R, Smith RM, Park BJ, Jarvis JN, Govender NP, Chiller TM (2017). Global burden of disease of HIV-associated cryptococcal meningitis: an updated analysis. Lancet Infect Dis.

[3] Li M, Chen Z, Xu L, Gan Z, Peng F, Liu J (2019). A comparison of the clinical characteristics and outcomes of cryptococcal meningitis in HIV-negative individuals with and without immunosuppression. Neurologist.

[4] Lofgren S, Abassi M, Rhein J, Boulware DR (2017). Recent advances in AIDS-related cryptococcal meningitis treatment with an emphasis on resource limited settings. Expert Rev Anti Infect Ther.

[5] Williamson PR, Jarvis JN, Panackal AA, Fisher MC, Molloy SF, Loyse A (2017). Cryptococcal meningitis: epidemiology, immunology, diagnosis and therapy. Nat Rev Neurol.

[6] Perfect JR, Dismukes WE, Dromer F, Goldman DL, Graybill JR, Hamill RJ (2010). Clinical practice guidelines for the management of cryptococcal disease: 2010 update by the infectious diseases society of America. Clin Infect Dis.

[7] Saag MS, Graybill RJ, Larsen RA, Pappas PG, Perfect JR, Powderly WG (2000). Practice guidelines for the management of cryptococcal disease. Infectious Diseases Society of America. Clin Infect Dis.

[8] Skipper C, Abassi M, Boulware DR (2019). Diagnosis and management of central nervous system cryptococcal infections in HIV-infected adults. J Fungi (Basel).

[9] Qu J, Zhou T, Zhong C, Deng R, Lü X (2017). Comparison of clinical features and prognostic factors in HIV-negative adults with cryptococcalmeningitis and tuberculous meningitis: a retrospective study. BMC Infect Dis.

[10] Anekthananon T, Manosuthi W, Chetchotisakd P, Kiertiburanakul S, Supparatpinyo K, Ratanasuwan W (2011). Predictors of poor clinical outcome of cryptococcal meningitis in HIV-infected patients. Int J STD AIDS.

[11] Ming DK, Harrison TS (2017). Cryptococcal meningitis. Br J Hosp Med (Lond).

[12] Chayakulkeeree M, Wangchinda P (2014). Clinical characteristics and outcomes of patients with cryptococcal meningoencephalitis in a resource-limited setting. J Med Assoc Thail.

[13] Jarvis JN, Bicanic T, Loyse A, Namarika D, Jackson A, Nussbaum JC (2014). Determinants of mortality in a combined cohort of 501 patients with HIV-associated Cryptococcal meningitis: implications for improving outcomes. Clin Infect Dis.

[14] Skripuletz T, Schwenkenbecher P, Pars K, Stoll M, Conzen J, Bolat S (2014). Importance of follow-up cerebrospinal fluid analysis in cryptococcal meningoencephalitis. Dis Markers.

[15] Liu ZY, Wang GQ, Zhu LP, Lyu XJ, Zhang QQ, Yu YS (2018). Expert consensus on the diagnosis and treatment of cryptococcal meningitis. Zhonghua Nei Ke Za Zhi.

[16] Tsai WC, Lien CY, Lee JJ, Hsiao WC, Huang CR, Tsai NW (2019). The clinical characteristics and therapeutic outcomes of cryptococcal meningitis in elderly patients: a hospital-based study. BMC Geriatr.

[17] Fuentes E, Fuentes M, Alarcón M, Palomo I (2017). Immune system dysfunction in the elderly. An Acad Bras Cienc.

[18] Yuchong C, Fubin C, Jianghan C, Fenglian W, Nan X, Minghui Y (2012). Cryptococcosis in China (1985-2010): review of cases from Chinese database. Mycopathologia.

[19] Mayer-Barber KD, Barber DL (2015). Innate and adaptive cellular immune responses to *mycobacterium tuberculosis* infection. Cold Spring Harb Perspect Med.

[20] Okoye AA, Picker LJ (2013). CD4(+) T-cell depletion in HIV infection: mechanisms of immunological failure. Immunol Rev.

[21] Huffnagle GB, Lipscomb MF, Lovchik JA, Hoag KA, Street NE (1994). The role of CD4+ and CD8+ T cells in the protective inflammatory response to a pulmonary cryptococcal infection. J Leukoc Biol.

[22] Zhong YH, Tan F, Li M, Liu J, Wang X, Yuan Y (2014). Comparisons of presentations and outcomes of cryptococcal meningitis between patients with and without hepatitis B virus infection. Int J Infect Dis.

[23] Lu CH, Chang WN, Chang HW, Chuang YC (1999). The prognostic factors of cryptococcal meningitis in HIV-negative patients. J Hosp Infect.

[24] Nau R, Sörgel F, Eiffert H (2010). Penetration of drugs through the blood-cerebrospinal fluid/blood–brain barrier for treatment of central nervous system infections. Clin Microbiol Rev.

[25] Concha-Velasco F, González-Lagos E, Seas C, Bustamante B (2017). Factors associated with early mycological clearance in HIV-associated cryptococcal meningitis. PLoS One.

[26] Jarvis JN, Casazza JP, Stone HH, Meintjes G, Lawn SD, Levitz SM (2013). The phenotype of the Cryptococcus-specific CD4+ memory T-cell response is associated with disease severity and outcome in HIV-associated cryptococcal meningitis. J Infect Dis.

[27] Brouwer AE, Rajanuwong A, Chierakul W, Griffin GE, Larsen RA, White NJ (2004). Combination antifungal therapies for HIV-associated cryptococcal meningitis: a randomised trial. Lancet.

[28] Xu L, Liu J, Zhang Q, Li M, Liao J, Kuang W (2018). Triple therapy versus amphotericin B plus flucytosine for the treatment of non-HIV- and non-transplant-associated cryptococcal meningitis: retrospective cohort study. Neurol Res.

[29] Zhao HZ, Wang RY, Wang X, Jiang YK, Zhou LH, Cheng JH (2018). High dose fluconazole in salvage therapy for HIV-uninfected cryptococcal meningitis. BMC Infect Dis.

[30] Scriven JE, Graham LM, Schutz C, Scriba TJ, Wilkinson KA, Wilkinson RJ (2017). The CSF immune response in HIV-1-associated Cryptococcal meningitis: macrophage activation, correlates of disease severity, and effect of antiretroviral therapy. J Acquir Immune Defic Syndr.

[31] Siddiqui AA, Brouwer AE, Wuthiekanun V, Jaffar S, Shattock R, Irving D (2005). IFN-gamma at the site of infection determines rate of clearance of infection in cryptococcal meningitis. J Immunol.

[32] Retini C, Vecchiarelli A, Monari C, Bistoni F, Kozel TR (1998). Encapsulation of Cryptococcus Neoformans with glucuronoxylomannan inhibits the antigen-presenting capacity of monocytes. Infect Immun.

